# The direct healthcare costs associated with psychological distress and major depression: A population-based cohort study in Ontario, Canada

**DOI:** 10.1371/journal.pone.0184268

**Published:** 2017-09-05

**Authors:** Maria Chiu, Michael Lebenbaum, Joyce Cheng, Claire de Oliveira, Paul Kurdyak

**Affiliations:** 1 Institute for Clinical Evaluative Sciences, Toronto, Ontario, Canada; 2 Institute of Health Policy, Management and Evaluation, University of Toronto, Toronto, Ontario, Canada; 3 Centre for Addiction and Mental Health, Toronto, Ontario, Canada; Boston University School of Public Health, UNITED STATES

## Abstract

The objective of our study was to estimate direct healthcare costs incurred by a population-based sample of people with psychological distress or depression. We used the 2002 Canadian Community Health Survey on Mental Health and Well Being and categorized individuals as having psychological distress using the Kessler-6, major depressive disorder (MDD) using DSM-IV criteria and a comparison group of participants without MDD or psychological distress. Costs in 2013 USD were estimated by linking individuals to health administrative databases and following them until March 31, 2013. Our sample consisted of 9,965 individuals, of whom 651 and 409 had psychological distress and MDD, respectively. Although the age-and-sex adjusted per-capita costs were similarly high among the psychologically distressed ($3,364, 95% CI: $2,791, $3,937) and those with MDD ($3,210, 95% CI: $2,413, $4,008) compared to the comparison group ($2,629, 95% CI: $2,312, $2,945), the population-wide excess costs for psychological distress ($441 million) were more than twice that for MDD ($210 million) as there was a greater number of people with psychological distress than depression. We found substantial healthcare costs associated with psychological distress and depression, suggesting that psychological distress and MDD have a high cost burden and there may be public health intervention opportunities to relieve distress. Further research examining how individuals with these conditions use the healthcare system may provide insight into the allocation of limited healthcare resources while maintaining high quality care.

## Introduction

Depression is an important public health concern that impacts approximately 350 million people worldwide [1] and is the third leading cause of disability globally [2]. In the United States, approximately 19.2% of adults experienced a major depressive episode at some point in their lives, while in Canada, the prevalence was 11.3% [3,4]. In addition to people with major depressive disorder (MDD), there are individuals who exhibit psychological distress, a more prevalent nonspecific condition characterized by symptoms of depression, anxiety and related somatic symptoms [5]. Psychological distress is associated with low socioeconomic status, unemployment, poor health and increased risk of mortality [6,7]. Despite the increased health burden of depression, few population-based cohort studies have examined the costs associated with major depression and far fewer studies have examined costs related to psychological distress [8].

Previous costing studies for depression have focused on patients in primary care or psychiatric care settings rather than population-based samples, which may be more representative of all people with depression, particularly those who do not seek health services [9–11]. Many studies have also estimated costs with limited adjustment for confounding factors, such as age, sex, income, lifestyle risk factors, and somatic illnesses [10,12,13]. Moreover, previous studies examining the cost of psychological distress did not have an MDD group for cost comparison and often relied on self-reported healthcare utilization [14,15]. Finally, there is insufficient evidence on which healthcare sectors are most associated with depression and psychological distress.

The primary objective of this study was therefore to determine the direct healthcare costs associated with psychological distress and major depression in Ontario. The secondary objectives were to understand how these costs vary by healthcare sector and what proportion of total costs were for mental health and addictions (MHA) versus non-MHA related healthcare services.

## Methods

### Study population

We used the Ontario sample of the Canadian Community Health Survey on Mental Health and Well Being, cycle 1.2 (CCHS 1.2) conducted by Statistics Canada in 2002 to select our study cohort. The CCHS 1.2 is a nationally representative community mental health survey of individuals aged 15 years or older living in private dwellings. The survey excluded individuals living on Indian Reserves and on Crown Lands, institutional residents, full-time members of the Canadian Forces, and residents of certain remote regions (98% coverage; 77% response rate) [16].

Using individual encrypted health card numbers, we anonymously linked survey respondents to multiple health administrative databases housed at the Institute for Clinical Evaluative Sciences (ICES) (S1 Table). We excluded respondents with invalid health card numbers or those without eligibility at baseline or a year prior which were required for data linkage and ascertaining covariates. Given our focus on psychological distress and depression, we excluded respondents with a hospitalization for bipolar disorder or schizophrenia/schizoaffective disorder in the past 5 years, as well as those who self-reported schizophrenia, any other psychoses, past year mania, dysthymia, or eating disorders (see S1 Table for codes). Given the cohort was recruited in 2002, the year ICD10 was implemented in Ontario Canada, parts of the 5-year lookback period used both ICD10 and ICD9 codes.

### Definitions of major depression, psychological distress and comparison groups

The CCHS 1.2 used the version of the World Health Organization (WHO) World Mental Health Composite International Diagnostic Interview (WMH-CIDI) [16] designed to assess the prevalence and burden of mental illness based on the definitions and criteria of the Diagnostic and Statistical Manual of Mental Disorders, 4^th^ edition (DSM-IV) systems [16].

In our study, the major depressive disorder (MDD) group included respondents who met the criteria for past 12-month MDD [16]. Among those who did not meet the criteria for MDD, those who scored 8 or more out of 24 on the Kessler 6 (K6) scale [17] were included in the psychological distress group. The K6 has been shown to be an effective screening instrument for psychological distress in the general population [7,18]. The remaining individuals comprised the comparison group.

### Cost estimations

#### Cost methodology

Costs for hospitalizations, ED visits, and outpatient visits were captured within health administrative databases (S1 Table). All billings by psychiatrists and MHA visits billed by primary care physicians were considered MHA costs. We used a validated algorithm by Steele et al. [19] to identify MHA visits billed by primary care physicians (i.e. family physicians and general practitioners). All other outpatient physician billings were considered non-MHA costs. Costs for hospitalizations or ED visits that were coded with a primary diagnosis of ICD-10 F00-F99 were considered MHA, while all other codes were considered non-MHA. All costs accrued in the Ontario Mental Health Reporting System were considered MHA costs. Other healthcare costs included: drug costs for those aged 65 years or over, other hospital, ambulatory care, long-term care, home care and devices (S2 Table). All costs were estimated using costing methods previously described in guidelines for person level costing [20].

#### Per-capita costs

For each individual, we estimated costs incurred from the survey interview date (2002) to the earliest of death, loss of healthcare eligibility or March 31, 2013 (end of study period), such that maximum follow-up was approximately 11 years. All costs were divided by the total length of follow-up to estimate annualized costs and inflated to 2013 values. These were then presented in United States Dollars (USD) using the OECD defined Canadian Dollar (CAD)-USD Purchasing Power Parities averaged over the study time period (1 CAD = 0.82 USD) [21]. We estimated the mean overall costs for each study group by both healthcare sector (i.e. physician, ED, hospital, and other) and MHA status (MHA spending, non-MHA spending).

### Statistical analysis

For outcomes with non-zero costs (i.e. Total Outpatient, All Costs, All Non-MHA Costs), we used a generalized linear model with a gamma distribution and a log link. For outcomes that included both zero and non-zero outcomes (i.e. all other cost categories), we used a two-part model, where the first part models whether costs occurred using a probit model and the second part models the level of costs among those with non-zero costs, conditional on incurring costs using a generalized linear model with a gamma distribution and a log link [22]. The choice of the distribution for the second part was based on the results of the modified Park test [22,23]. We modelled each sector of costs (i.e. Outpatient, ED, hospital, drug, ambulatory, and each type of other costs) and total costs by MHA status (MHA costs, non-MHA costs) separately.

Results from the two-part models are shown as predicted per-capita costs and per-capita cost differences using marginal standardization to estimate the predicted costs [24]. Per-capita excess costs were calculated by comparing costs in each exposure group to the comparison group. Excess cost was defined as the difference between the cost in each exposure group and the cost in the comparison group, to reflect the cost above and beyond those expected in the comparison group. Statistical tests compared the costs in each exposure group relative to the comparison group and compared the two exposure groups. All results were weighted by the survey weights to allow generalizability to the overall Ontario population and bootstrap methods were used to calculate confidence intervals. Analyses were conducted using STATA v13.1.

We estimated unadjusted, age- and sex-adjusted, and multivariable-adjusted costs. Multivariable-adjusted models included covariates measured at baseline that are thought to be associated with costs and mental health. Most covariates were derived from the survey responses including: age, sex, marital status (married/common law vs other), residence (urban vs rural), ethnicity (white vs non-white), immigrant status (immigrant vs non-immigrant) [25], current smoking (yes/no) [26], physical inactivity (i.e., energy expenditure <1.5 kcal/kg/day) [27], body-mass index [27]. The remaining covariates were derived from administrative databases including area-level low income status (quintiles 1–2 vs 3–5) [28] measured at the level of the dissemination area and five measures of baseline medical comorbidities ascertained using validated algorithms [29–34]; i.e. cardiovascular diseases, cancer, respiratory conditions, hypertension and diabetes.

To estimate the weighted number of individuals within each group we summed up the sample weights of all respondents within each group. To calculate the age- and sex-adjusted population-wide costs or population-wide excess costs, we multiplied the weighted sample size within each group by the age- and sex-adjusted per-capita cost or per-capita excess costs within each study group.

## Results

From a total of 10,662 initial survey respondents, we excluded 356 respondents with other serious mental illnesses and 145 individuals without healthcare eligibility at baseline and a year prior to baseline, and 6 individuals withdata errors (i.e. 4 with a survey date outside recruitment window, 2 with a death data prior to the survey date suggesting a possible data linkage error). There was a total of 190 (1.7%) missing observations which were excluded from the analysis. Our final sample consisted of 9,965 individuals: 651 with psychological distress, 409 with MDD and 8,905 comparison group individuals (Table 1). The weighted prevalence of psychological distress and MDD was 6.6% and 3.9%, respectively. Median follow-up time was 10.6 years in all exposure groups. Individuals with psychological distress and MDD were significantly younger than the comparison group (40.8 and 39.3 vs. 44.3 years, respectively) and were less likely to be married or in common-law relationships (p<0.001) (Table 1).

**Table 1:** Sociodemographic and risk factor profile of those with psychological distress, major depressive disorder (MDD) and the comparison group at baseline (n = 9,965)^a^.

	Comparison Group (CG)	Psychological distress (PD)	*P*-values^f^ (PD vs. CG)	MDD	*P*-values^f^ (MDD vs. CG)	*P*-values (MDD vs. PD)
Unweighted N	8905	651		409		
Mean age (years)	44.3	40.8	<0.001	39.3	<0.001	0.24
Male sex	50.6	46.4	0.12	36.9	<0.001	0.053
Married/Common Law	65.0	55.6	<0.001	48.5	<0.001	0.11
Urban residence	82.8	86.9	0.09	80.7	0.57	0.15
Income quintile 1–2	40.3	47.1	0.018	42.0	0.63	0.30
Non-white ethnicity	21.5	25.9	0.10	16.2	0.21	0.055
Non-immigrant	68.0	64.2	0.20	71.9	0.36	0.13
Body-mass index (BMI), mean (median)^b^	25.5 (24.9)	25.4 (24.8)	0.59	25.1 (23.8)	0.31	0.62
Cigarette smoker	26.1	31.6	0.01	46.6	<0.001	<0.001
Physical inactivity^c^	45.4	57.5	<0.001	56.9	0.003	0.89
Cardiovascular diseases^d^	2.4	3.7	0.12	1.4	0.31	0.09
Cancer	3.9	2.6	0.24	1.7	0.11	0.50
Respiratory conditions^e^	13.2	15.0	0.32	18.2	0.02	0.30
Hypertension	20.7	18.5	0.26	14.4	0.01	0.17
Diabetes	6.0	8.1	0.10	5.2	0.59	0.16

^a^ % unless otherwise specified; all values weighted by sample weights, bootstrap methods used to determine *P*-values

^b^ BMI was based on self-reported height and weight in kg/m^2^

^c^ Physical inactivity includes energy expenditure of <1.5 kcal/kg/day

^d^ Cardiovascular diseases include acute myocardial infarction and congestive heart failure

^e^ Respiratory conditions include chronic obstructive pulmonary disease and asthma

^f^ *P*-values generated based on comparisons to the comparison group; t-tests for continuous variables and chi-square test for binary variables

### Average annual per-capita direct healthcare costs

Our findings were consistent across unadjusted, age- and sex-adjusted, and multivariate adjusted models; below, we focus on age- and sex-adjusted results.

The annual per-capita total costs were similar between the psychological distress ($3,364, 95% CI: $2,791, $3,937) and MDD ($3,210, 95% CI: $2,413, $4,008) groups and both had higher costs than the comparison group ($2,629, 95% CI: $2,312, $2,945) (Table 2). The psychological distress and MDD groups incurred higher healthcare costs in all sectors examined (i.e. outpatient, ED, hospital and other healthcare sectors) (Table 3). The MDD group had significant excess ED costs ($41, 95% CI: $7, $74) and larger but non-significant excess total costs ($582, 95% CI: -$360, $1,524) compared to the comparison group. When psychological distress and MDD groups were compared, outpatient and ED excess costs were similar (excess per-capita cost < $35 and p > 0.60 in both cases). Cost-differences for hospital, other non MHA, total, and total non-MHA were also not significantly different between depressed and psychological distressed individuals (p > 0.19 in all cases). We observed a dose-response relationship across psychological distress, MDD, and increased MHA costs; both were significantly greater than the comparison group (excess per-capita cost: $68, p = 0.04 and $364, p = 0.003, respectively) and MHA costs were significantly greater among depressed individuals compared to those with psychological distress ($296, p = 0.02)(Table 3). As a percentage of total costs, MHA-related costs were greater among the MDD group (13.5%) relative to the psychological distress group (4.1%), which interestingly was similar to thecomparison group (2.7%) (Table 2). Although MHA costs were lower in the psychological distress group relative to the MDD group, they incurred greater non-MHA costs than the MDD group (Table 3).

**Table 2:** Per-capita absolute costs incurred by those with psychologically distressed, major depressive disorder (MDD) and comparison group per person year (prices in 2013 USD)^a^.

	Comparison Group	Psychological distress	MDD
**Outpatient**			
Unadjusted^d^	790 (721–858)	894 (798–989)	850 (731–969)
Age-sex adjusted^d^	809 (725–893)	979 (869–1,088)	944 (802–1,085)
Multivariable adjusted^d^	817 (743–891)	979 (877–1,081)	956 (831–1,082)
**Emergency Department**			
Unadjusted	99 (89–110)	135 (113–156)	118 (96–139)
Age-sex adjusted	97 (85–108)	139 (118–160)	137 (109–165)
Multivariable adjusted	100 (88–111)	130 (113–148)	129 (103–154)
**Hospital**			
Unadjusted	942 (831–1,052)	975 (740–1,210)	1,014 (586–1,441)
Age-sex adjusted	880 (756–1,004)	1,011 (770–1,253)	1,139 (661–1,616)
Multivariable adjusted	928 (785–1,072)	988 (778–1,197)	1,122 (712–1,532)
**Other healthcare**^b^			
Unadjusted	874 (795–953)	1,337 (973–1,700)	840 (502–1,179)
Age-sex adjusted	786 (671–901)	1,199 (699–1,699)	819 (516–1,122)
Multivariable adjusted	902 (772–1,032)	1,388 (769–2,007)	839 (567–1,111)
**All costs**			
Total			
Unadjusted^d^	2,704 (2,493–2,916)	3,340 (2,770–3,910)	2,822 (2,086–3,557)
Age-sex adjusted^d^	2,629 (2,312–2,945)	3,364 (2,791–3,937)	3,210 (2,413–4,008)
Multivariable adjusted^c^^d^	2,831 (2,529–3,133)	3,411 (2,856–3,965)	3,223 (2,502–3,944)
Mental Health or Addictions (MHA)			
Unadjusted	73 (53–93)	114 (65–163)	359 (171–548)
Age-sex adjusted	70 (52–88)	138 (74–203)	434 (192–677)
Multivariable adjusted^c^	72 (54–91)	132 (75–189)	487 (184–789)
Non-MHA			
Unadjusted^d^	2,631 (2,421–2,842)	3,226 (2,657–3,796)	2,462 (1,774–3,150)
Age-sex adjusted^d^	2,567 (2,248–2,886)	3,214 (2,646–3,782)	2,667 (1,996–3,338)
Multivariable adjusted^c^^d^	2,770 (2,464–3,077)	3,269 (2,716–3,822)	2,628 (2,056–3,201)
% of total costs that are for MHA-related health services			
Unadjusted	2.7	3.4	12.7
Age-sex adjusted	2.7	4.1	13.5
Multivariable adjusted^c^	2.5	3.9	15.1

^a^ All values weighted by sample weights, bootstrap methods used to determine 95% confidence intervals

^b^ Outpatient prescriptions for adults age 65 and older, non-hospital based residential care, ambulatory care, home care, medical devices

^c^ Adjusted for age, sex, marital status, urban vs rural residence, area-level low income status (quintiles 1–2 vs 3–5), white vs non-white ethnicity, immigrant status, smoking, physical inactivity (energy expenditure <1.5 kcal/kg/day) body-mass index, cardiovascular diseases, cancer, respiratory conditions, hypertension and diabetes.

^d^ One part models were used, all other models were two-part models

**Table 3:** Age- and sex-adjusted per-capita excess costs incurred by those with psychological distress and major depressive disorder (MDD) compared to the comparison group per person year (prices in 2013 USD)^a^.

	Psychological distress		MDD	
	Difference (95% CI)	p-value	Difference (95% CI)	p-value
**Outpatient**				
Unadjusted^e^	104 (-17–226)	0.093	61 (-77–198)	0.389
Age-sex adjusted^e^	170 (16–324)^b^	0.031	135 (-56–326)	0.165
Multivariable adjusted^e^	162 (43–281)^b^	0.008	139 (4–275)^b^	0.044
**Emergency Department**				
Unadjusted	36 (12–60)^b^	0.004	19 (-6–43)	0.132
Age-sex adjusted	43 (17–68)^b^	0.001	41 (7–74)^b^	0.018
Multivariable adjusted	31 (12–50)^b^	0.001	29 (4–55)^b^	0.024
**Hospital**				
Unadjusted	34 (-228–295)	0.801	72 (-379–523)	0.754
Age-sex adjusted	131 (-176–439)	0.403	259 (-272–789)	0.34
Multivariable adjusted	59 (-188–306)	0.638	194 (-226–614)	0.365
**Other healthcare**^c^				
Unadjusted	463 (91–834)^b^	0.015	-34 (-382–315)	0.849
Age-sex adjusted	413 (-129–954)	0.135	33 (-316–381)	0.855
Multivariable adjusted	486 (-162–1,134)	0.142	-62 (-364–239)	0.684
**All costs**				
Total				
Unadjusted^e^	636 (20–1,252)^b^	0.043	117 (-663–897)	0.768
Age-sex adjusted^e^	735 (27–1,444)^b^	0.042	582 (-360–1,524)	0.226
Multivariable adjusted^d^^e^	580 (-71–1,230)	0.081	392 (-362–1,147)	0.308
Mental Health or Addictions (MHA)				
Unadjusted	41 (-13–94)	0.136	287 (98–475)^b^	0.003
Age-sex adjusted	68 (4–133)^b^	0.038	364 (125–604)^b^	0.003
Multivariable adjusted^d^	60 (7–113)^b^	0.027	414 (118–711)^b^	0.006
Non-MHA				
Unadjusted^e^	595 (-20–1,210)	0.058	-169 (-901–563)	0.65
Age-sex adjusted^e^	647 (-54–1,348)	0.071	100 (-718–918)	0.81
Multivariable adjusted^d^^e^	499 (-150–1,148)	0.132	-142 (-748–464)	0.646

^a^ All values weighted by sample weights, bootstrap methods used to determine 95% confidence intervals

^b^ Significant results (i.e. 95% confidence interval for the difference does not cross $0)

^c^ Outpatient prescriptions for adults age 65 and older, non-hospital based residential care, ambulatory care, home care, medical devices

^d^ Adjusted for age, sex, marital status, urban vs rural residence, area-level low income status (quintiles 1–2 vs 3–5), white vs non-white ethnicity, immigrant status, smoking, physical inactivity (energy expenditure <1.5 kcal/kg/day) body-mass index, cardiovascular diseases, cancer, respiratory conditions, hypertension and diabetes.

^e^ One part models were used, all other models were two-part models

### Population-wide costs

**Fig 1:**
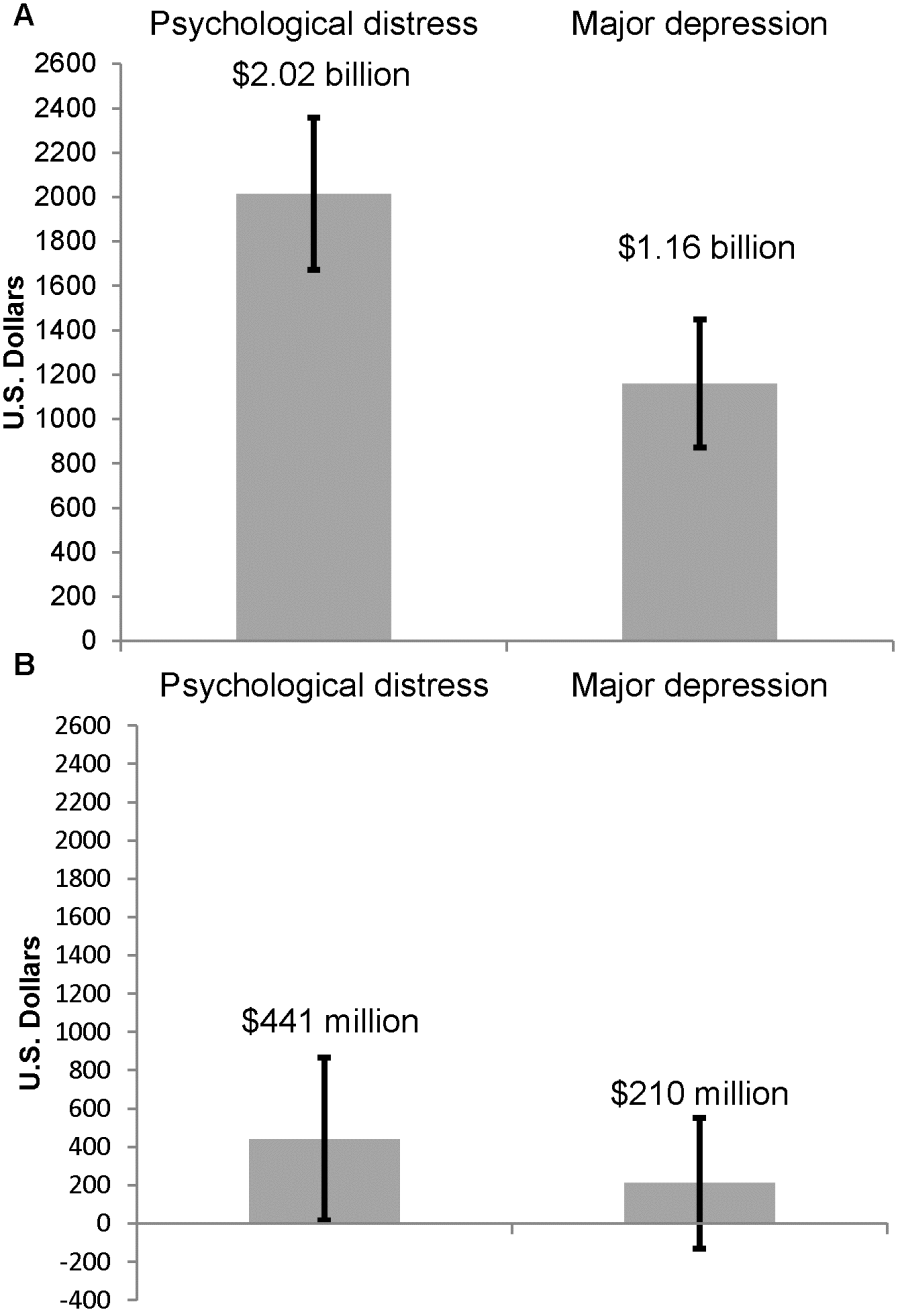
Population-wide annual absolute (Panel A) and excess (Panel B) direct health care costs (2013 USD) incurred by people with psychological distress and depression in Ontario, Canada^a^. ^a^ All values weighted by sample weights. Analyses were adjusted for age and sex. Bootstrap methods were used to determine the 95% confidence intervals (i.e. the 2.5th and 97.5th percentiles of 500 bootstrap estimates).

At the population level, 599,047 were psychologically distressed, 361,389 had MDD, and 8,260,184 individuals had neither condition. The age-and-sex adjusted annual absolute healthcare costs were $2.0 billion for the psychologically distressed population and $1.2 billion for the MDD population (Fig 1 and S3 Table). This represented an excess of $441 (95% CI: $16, $865) million for the psychologically distressed population and $210 (95% CI: -$130, $551) million for the MDD population compared to the comparison group (Fig 1 and S3 Table) as there was a greater number of people with psychological distress than depression.

## Discussion

To our knowledge, this study is the first population-based cohort study to estimate the direct healthcare costs associated with individuals with psychological distress who do not meet the criteria for depression, compared to those with MDD and the comparison group with neither condition. Our study found that individuals with psychological distress and MDD incurred greater direct healthcare costs per capita than the comparison group, with significantly large excess outpatient and ED costs. Although on a per-capita basis, individuals with elevated distress have similar costs to those with depression, their annual population-wide costs were almost twice the cost burden associated with depression. Despite similar costs, only 4% of the costs incurred by those with psychological distress are associated with MHA-related health services compared to 14% among individuals with depression, suggesting that individuals with psychological distress may not be identified or treated for their mental health condition. We acknowledge that some of the non-MHA costs are due to higher chronic conditions, although at baseline none of the major chronic diseases were significantly more prevalent among individuals with psychological distress than in the comparison group and adjustment for chronic conditions resulted in minimal changes to the results. Our findings emphasize the need to better understand psychological distress and reasons for these elevated healthcare costs.

Very few previous studies have investigated the association between psychological distress and healthcare costs [8,14,15,35,36]; most have been based on the US Medical Expenditure Panel Survey and none included a validated depressed group for comparison [8,14,15]. These studies have consistently shown that psychological distress is associated with significant healthcare expenditures compared to those without psychological distress [8,14,15,35,36]. Results from a contemporaneous survey in the U.S. showed psychological distress was significantly associated with $1,735 higher total per capita excess expenditures, $1,000 higher than our estimate. This may be driven by differences in methodology (e.g. use of self-reported utilization to derive costs, different cut-off value on the K6 scale) and differences in health systems [14].

A primary finding of our study was that individuals with psychological distress cost the healthcare system a similar amount on a per-capita basis as those with MDD. Our findings are similar to results from a US study, which showed no statistically significant differences in costs between elderly patients with subthreshold depressive symptoms, a similar condition to psychological distress, and those with DSM-IV depressive disorders [9]. However, a Dutch study found the medical cost of minor depression to be considerably lower than the medical cost of major depression [37]. The Dutch study derived costs from self-reported medical consumption and may be subject to respondent bias, while our estimates were derived from a large population-based cohort using objective healthcare cost data from administrative databases.

Another key finding is the dose-response gradient in mental health costs across the study groups. In a study by Kleine-Budde et al. [38], mean annual depression-specific healthcare costs for persons with severe depression were twice as high as those for individuals with moderate depression and five times higher than those with mild depression. This dose-response relationship was also observed for total costs among those with increasing levels of psychological distress [15]. Moreover, our findings on sector-specific costs were comparable to results found in a study by Katon et al. [9], such that outpatient costs were significantly higher in both the minor and major depression groups compared to the comparison group. ED visit costs were significantly higher in both groups in our study and although elevated in both groups in the Katon et al. [9] study, it was only significant among those with minor depression. In our study, we also observed that non-MHA costs were higher for those with psychological distress than MDD. This can be attributed to the fact that individuals suffering from psychological distress often seek help for their unexplained physical symptoms, but may not be diagnosed or treated as mental health conditions [39].

Our study showed that the per-capita excess costs of psychological distress and MDD were $897 and $709 respectively. A systematic review by Luppa et al. [40] demonstrated that excess direct costs among the subclinically depressed compared to the comparison group ranged from $789 to $2,263, whereas excess costs for depressed ranged from $324 to $5,871 per person per year. Our estimates of costs lie on the lower end of this range perhaps because we could not include the cost of drugs, laboratory tests or non-physician, ED and hospital-based services. In general, it is difficult to compare costs across jurisdictions as there is often variability in health systems, available services, populations and patterns of health service utilization [40]; this underscores the value of Canadian-specific cost estimates for these important psychological conditions.

This study has several strengths. First, unlike previous studies [14,36], the linkage of our study cohort to several health administrative databases allowed us to directly measure prospective longitudinal costs for the full follow-up period for almost all individuals without relying on personal retrospective recall of healthcare utilization. Second, MDD was determined through a validated structured diagnostic interview (CIDI DSM-IV) [41–43] and our measure of psychological distress was determined using the K6 scale which has been shown to be useful for screening purposes [18,44]. Third, unlike previous studies that often estimated costs for MDD from clinical samples, our study used a population-based sample and survey weights were applied in all models allowing generalizable cost estimates enabling the calculation of per-capita and population-wide costs. Fourth, the universal healthcare system in Ontario reduces the issue of access to physician and hospital care for patients with limited financial means for paying for care. Finally, this study was able to control for important covariates at baseline, including chronic medical conditions. Nevertheless, we acknowledge that there may be additional factors that jointly influence depression or psychological distress and healthcare costs that we are unable to control for.

This study also has some limitations that need to be considered. First, the CCHS excluded certain vulnerable populations [16], some of whom may have elevated burden of psychiatric disorders, therefore our cost estimates are likely conservative. Second, given that MDD or distress status and covariates were only assessed at baseline and costs were assessed over an 11-year follow-up period, we were unable to appropriately account for time. It is conceivable that individuals may have moved between exposure groups during the 11-year follow-up period or major (ecological) events have occurred during the 11-year follow-up period that may have affected costs. Third, we were not able to capture drug costs for adults under age 65 years or community mental health and addictions services. Moreover, costs for outpatient psychological counselling were not captured as they are largely covered through employer-based insurance or paid out-of-pocket. All of these cost categories (i.e. drug, community mental health and addiction services, counselling) are likely to be greater among individuals with psychological problems and therefore resulting in an underestimate of the total healthcare costs among those with psychological distress and depression. This might suggest that our estimates of healthcare costs are likely conservative. However, a prior review suggested that only a small proportion of the excess healthcare costs among depressed patients are accounted for by depression treatment costs suggesting the underestimate is not substantial [45]. Nevertheless, the universal healthcare system in Ontario and the availability of data from most major healthcare sectors at the patient-level allowed us to capture the majority of direct healthcare costs. We were also limited in determining whether the care received was for disease management or preventive care. Given higher ED and primary care costs, this may signal that despite elevated primary care use, these individuals are not receiving adequate mental health care in the primary care setting to fully address their needs and prevent ED visits. Future work is necessary to examine the quality of primary mental health care received and whether unmet needs for care are driving higher emergency department visits.

## Conclusions

In this population-based study, we found that people with psychological distress and major depression incurred similar per-capita direct healthcare costs and these costs were greater than those without either condition. These two groups differed in the way the totals were reached, with depressed individuals incurring higher mental health and addiction-related costs and psychologically distressed individuals incurring higher non-mental health and addiction costs. Given the high prevalence of psychological distress, at the population level, this group incurred almost twice the healthcare costs compared to individuals with major depression, despite not meeting the clinical cut-off for MDD. The elevated rate of contact with the healthcare system among patients with psychological distress provides an opportunity for them to be identified and receive mental healthcare. Further research is needed to examine whether identifying and treating psychological distress will result in short- or long-term cost savings.

## Supporting information

S1 Table ICD9 and ICD10 diagnostic codes. (DOCX)

S2 Table Administrative databases used to capture costs by healthcare sector. (DOCX)

S3 Table Age- and sex-adjusted population-wide healthcare cost incurred by those with psychological distress, major depressive disorder (MDD) and the comparison group, by sector (in millions of 2013 USD). (DOCX)
