# Supplementary material for: The direct healthcare costs associated with psychological distress and major depression: A population-based cohort study in Ontario, Canada
Source: PLoS One. 2017 Sep 5;12(9):e0184268. doi: 10.1371/journal.pone.0184268 (PMC5584795; doi:10.1371/journal.pone.0184268)
Supplement: S1 Table — (DOCX) [file pone.0184268.s001.docx]

**S1 Table:** ICD9 and ICD10 Diagnostic codes

| Condition | ICD9 | ICD10 |
| --- | --- | --- |
| Bipolar disorder | 2960 Manic Disorder  2961 Recurrent Manic Disorder  2964 Bipolar Affective Manic  2965 Bipolar Affective Depressed  2966 Bipolar Affective Mixed  2967 Bipolar Affective NOS  2968 Bipolar Other and unspecified | F30 Manic episode  F31 Bipolar affective disorder |
| Schizophrenia/schizoaffective disorder | 295 Schizophrenia / Schizoaffective disorders | F20 Schizophrenia  F25 Schizoaffective disorders |
