# Supplementary material for: The direct healthcare costs associated with psychological distress and major depression: A population-based cohort study in Ontario, Canada
Source: PLoS One. 2017 Sep 5;12(9):e0184268. doi: 10.1371/journal.pone.0184268 (PMC5584795; doi:10.1371/journal.pone.0184268)
Supplement: S2 Table — (DOCX) [file pone.0184268.s002.docx]

**S2 Table:** Administrative databases used to capture costs by healthcare sector.

| **Costs captured in this study** | **Database** |
| --- | --- |
| Outpatient | Ontario Health Insurance Plan (OHIP) |
| Emergency Department | National Ambulatory Care Reporting System (NACRS) |
| Hospital |  |
| All inpatient discharges prior to 2005, all discharges from non-mental health beds after 2005 | Canadian Institute for Health Information hospital Discharge Abstract Database (CIHI-DAD) |
| Inpatient admissions to mental health beds 2005 onwards | Ontario Mental Health Reporting System (OMHRS) |
| Other healthcare | |
| Outpatient prescriptions for adults age 65 and older | Ontario Drug Benefit (ODB) |
| Other Hospital: |  |
| Stays in complex continuing care facilities | Continuing Care Reporting System (CCRS) |
| Rehabilitation services | National Rehabilitation Reporting System (NRS) |
| Stays in long term care facilities | Long term care (CCRS) |
| Ambulatory care: Same day surgery, dialysis and cancer care clinics | National Ambulatory Care Reporting System (NACRS) |
| Home care | Ontario Home Care Administrative System (OHCAS) (pre March 31, 2005) / Home Care Database (HCD) (post March 31, 2005) |
| Medical devices | Assistive Devices Program (ADP) |
