# Supplementary material for: The direct healthcare costs associated with psychological distress and major depression: A population-based cohort study in Ontario, Canada
Source: PLoS One. 2017 Sep 5;12(9):e0184268. doi: 10.1371/journal.pone.0184268 (PMC5584795; doi:10.1371/journal.pone.0184268)
Supplement: S3 Table — (DOCX) [file pone.0184268.s003.docx]

**S3 Table:** Age- and sex-adjusted population-wide healthcare cost incurred by those with psychological distress, major depressive disorder (MDD) and the comparison group, by sector (in millions of 2013 USD).^a^

|  | Population-wide absolute costs^b^ | | Population-wide excess costs^c^ | |
| --- | --- | --- | --- | --- |
|  | **Psychological distress** | **MDD** | **Psychological distress vs the comparison group** | **MDD vs the comparison group** |
| Outpatient | 586 (521 – 652) | 341 (290 – 392) | 102 (10 – 194) | 49 (-20 – 118) |
| Emergency department | 83 (71 – 96) | 50 (39 – 60) | 26 (10 – 41) | 15 (3 – 27) |
| Hospital | 606 (461 – 751) | 412 (239 – 584) | 79 (-105 – 263) | 93 (-98 – 285) |
| Other healthcare | 718 (419 – 1018) | 296 (186 – 405) | 247 (-77 – 571) | 12 (-114 – 138) |
| Total | 2015 (1672 – 2358) | 1160 (872 – 1448) | 441 (16 – 865) | 210 (-130 – 551) |

^a^ Sample sizes used: Comparison Group (n=8,260,184), Psychological distress (n = 599,047), major depression (n = 361,389)

^b^ Population-wide absolute costs were calculated as follows: psychological distress group = per-capita cost in psychological distress group x N_Psychological distress_; major depression group = per-capita cost in major depression group x N_Major depression_

^c^ Population-wide excess costs were calculated as follows: psychological distress group = (per-capita cost in psychological distress group –per-capita cost among the comparison group) x N_Psychological Distress_; major depression group = (per-capita cost in major depression group –per-capita cost among the comparison group) x N_Major depression_; where N_Psychological Distress_ =599,047 and N_Major depression_ =361,389 the weighted sample sizes in the psychological distress and major depression groups, respectively.
